# A recombinant monoclonal-based Taenia antigen assay that reflects disease activity in extra-parenchymal neurocysticercosis

**DOI:** 10.1371/journal.pntd.0010442

**Published:** 2022-05-26

**Authors:** Madelynn Corda, Joshua Sciurba, Jiana Blaha, Siddhartha Mahanty, Adriana Paredes, Hector H. Garcia, Theodore E. Nash, Thomas B. Nutman, Elise M. O’Connell

**Affiliations:** 1 Laboratory of Parasitic Diseases, National Institute of Allergy and Infectious Diseases, Bethesda, Maryland, United States of America; 2 The Peter Doherty Institute for Infection and Immunity, University of Melbourne & The Royal Melbourne Hospital, Melbourne, Victoria, Australia; 3 Universidad Peruana Cayetano Heredia, Lima, Peru; Baylor College of Medicine, UNITED STATES

## Abstract

**Background:**

Antigen tests for diagnosis and disease monitoring in some types of neurocysticercosis (NCC) are useful but access to testing has been limited by availability of proprietary reagents and/or kits.

**Methods/Principal findings:**

Three previously identified IgM-secreting hybridomas whose IgM products demonstrated specificity to *Taenia solium* underwent variable heavy and light chain sequencing and isotype conversion to mouse IgG. Screening of these recombinantly expressed IgG anti-Ts hybridomas, identified one (TsG10) with the highest affinity to crude *Taenia* antigen. TsG10 was then used as a capture antibody in a sandwich antigen detection immunoassay in combination with either a high titer polyclonal anti-Ts antibody or with biotinylated TsG10 (termed TsG10*bt). Using serum, plasma, and CSF samples from patients with active NCC and those from NCC-uninfected patients, ROC curve analyses demonstrated that the TsG10-TsG10-*bt assay achieved a 98% sensitivity and 100% specificity in detecting samples known to be antigen positive and outperformed the polyclonal based assay (sensitivity of 93% with 100% specificity). By comparing levels of Ts antigen (Ag) in paired CSF (n = 10) or plasma/serum (n = 19) samples from well-characterized patients with extra-parenchymal NCC early in infection and at the time of definitive cure, all but 2 (1 from CSF and 1 from plasma) became undetectable. There was a high degree of correlation (r = 0.98) between the Ag levels detected by this new assay and levels found by a commercial assay. Pilot studies indicate that this antigen can be detected in the urine of patients with active NCC.

**Conclusions/Significance:**

A newly developed recombinant monoclonal antibody-based Ts Ag detection immunoassay is extremely sensitive in the detection of extra-parenchymal NCC and can be used to monitor the success of treatment in the CSF, serum/plasma and urine. The ability to produce recombinant TsG10 at scale should enable use of this antigen detection immunoassay wherever NCC is endemic.

**Clinical Trial Registration:**

ClinicalTrials.gov Identifiers: NCT00001205 - & NCT00001645.

## Author summary

Neurocysticercosis, an NTD caused by the helminth *Taenia solium*, is a major cause of neurologic symptoms in endemic areas. Diagnosis rests upon imaging of the central nervous system, which is not always available, and is not specific in many cases. Testing for *T*. *solium* antigen in the peripheral blood and cerebral spinal fluid of patients with a high burden of neurocysticercosis has already been shown to have a high utility in assisting in diagnosis and following over the course of therapy, particularly for extra parenchymal disease. Here we describe a capture ELISA assay (called TsG10) with high sensitivity and specificity for detecting *T*. *solium* antigen that was produced recombinantly and can be easily transitioned into a lateral flow assay for point of care testing. We also demonstrate the utility in following this antigen assay over the course of treatment for patients with extra-parenchymal disease. Additionally, we publish the antibody sequence for use by any lab with the interest and capabilities of performing this test, without the need for access to a hybridoma.

## Introduction

*Taenia solium*, the pork tapeworm, is endemic to most of Latin America, Asia, and sub-Saharan Africa, particularly where pigs are raised in close proximity to humans. The prevalence of infection in high-income countries is proportional to immigration from endemic regions. While neurocysticercosis (NCC) is a costly and significant problem in the US, totaling over 18,000 hospitalizations and greater than US $908 million between the years of 2003 and 2012, the prevalence is unknown [1]. Human infection with *T*. *solium* occurs following oral ingestion of eggs passed in human feces from a tapeworm carrier. The larvae can travel anywhere in the body, but once the parasite penetrates the central nervous system (CNS), the disease associated with infection is referred to as NCC. NCC is often categorized as parenchymal, ventricular, or subarachnoid (racemose) according to which space in the CNS the cysts occupy.

Currently, the diagnosis is most often made through the use of radiological imaging studies (i.e. computed tomography [CT] and/or magnetic resonance imaging [MRI]) to visualize the morphology, stage, and location of cysts in combination with serological assays for the detection of antibodies to *T*. *solium*. Since antibodies can persist for some time after an infection is cleared, detection of antibodies does not distinguish between active infection and a previous exposure to *T*. *solium*. Having biomarkers to follow over the course of treatment in subarachnoid NCC (SANCC) is particularly warranted given that radiographs rarely normalize following treatment [2]. Previous studies have demonstrated that *T*. *solium* DNA and antigen tend to be high in the peripheral blood and CSF during active disease and fall to undetectable levels with cure in subarachnoid and ventricular NCC [3]. While qPCR may not be practical in all settings, antigen detection is easily deployable in a wide variety of settings. Two distinct antigen tests have been most widely used; both these systems rely on monoclonal antibodies (Mabs), either HP10 [4] or B158/B60 [5], but their use has been limited either because of lack of available reagents or inability to acquire the commercial kit (e.g. ApDia). Here we demonstrate the utility of a new Taenia antigen assay using a recombinantly expressed TsG10 mouse IgG Mab. This assay has performance characteristics not unlike the assays previously described [4, 5]. However, the ability to produce recombinant TsG10 at scale and the availability of the sequence information for this antibody should enable use of this antigen detection immunoassay wherever NCC is endemic.

## Materials and methods

### Ethics statement

All patient samples with linked clinical data were enrolled on the National Institutes of Health Intramural IRB-approved clinical protocols “Natural History of Treated Neurocysticercosis and Long-Term Outcomes” (ClinicalTrials.gov Identifier NCT00001205) or “Evaluation, Treatment and Monitoring of Patients with a Known or Suspected Parasitic Infection” (NCT00001645). All patients provided written consent.

### Sample collection

Plasma, serum, urine, and CSF were collected from a well described cohort of neurocysticercosis patients enrolled on a natural history protocol. Samples collected for neurocysticercosis clinical monitoring by qPCR were also tested if there was excess sample after required testing. Healthy donor control sera used were from NIH blood bank donated sera. Each of these control sera have been shown to be seronegative in based on responses to 3 recombinant *T*. *solium* antigens described previously [6]. Deidentified putatively uninfected CSF that would have otherwise been thrown away was obtained from the clinical microbiology department after clinical testing were used as NCC negative CSF controls.

### Monoclonal antibody identification

Three hybridomas generating previously described monoclonal antibodies (mAb) to *T*. *solium* [7] were selected based on their production, antigen-binding specificity, and reactivity. These IgM antibodies, TsW5, TsW8, and TsW11, were shown to have the highest binding ability to the structural components of Taenia cysts [7].

### Monoclonal IgM to recombinant IgG antibody conversion

RNA was prepared from the TsW5, TsW8, and TsW11 hybridomas and the variable heavy and light chains were sequenced (ProMab Biotechnologies, Inc.). Full length IgG antibodies were expressed in HD293F cell (GenScript Inc, USA). Because the V_h_ and V_l_ sequences of TsW5 and TsW8 were identical, we expressed 2 distinct antibodies TsG10 (derived from IgM TSW5) and TsH12 (derived from TSW11).

### Antigen preparation

*Taenia crassiceps* (Tc) cysts (ORF strain) were extracted from infected mice, washed, and loaded into 2 mL Precellys tubes (Bertin Technologies S.A.S, cat no. SK38). Phosphate-buffered saline (PBS) with a protease inhibitor cocktail was added to bring volumes to the topmost demarcation on the tubes. Using the Precellys 24 (Bertin Instruments, #P000669-PR240-A), tubes were thrice homogenized for 20 seconds at 5000 revolutions per minute (RPM) and then centrifuged for 2 minutes at 2000 RPM. The aqueous phase was then removed and placed into a 50 mL tube (Fisher Scientific, #14-432-22) on ice. Another 1 mL of PBS with protease inhibitor was added to each Precellys tube for a second homogenization. This was repeated twice more for a total of 4 homogenizations. After the final homogenization, all remaining fluid was transferred to a a 50ml conical tube and sonicated on ice at 60 amps for 3 cycles of 60 seconds each with 60 seconds of rest in between. The fluid was transferred into a 125 mL Erlenmeyer flask and a magnetic stir bar was used to stir the solution overnight at 4°C. Finally, the product was centrifuged at 33745 relative centrifugal force (RCF) for 60 minutes at 4°C and the aqueous phase was decanted, filtered through a 45-um filter, aliquoted, and stored overnight at -20°C and then -80°C.

### Antibody selection

IgM and IgG analogues were compared with direct ELISAs. Antigen was diluted in PBS to 10ng/mL and six 2-fold dilutions were performed and used to coat 96-well microtiter plates (Immulon 4 HBX, Thermo Scientific) at 100ul/well. Plates were incubated overnight at 4°C. Following blocking and washing (see details below), the IgMs (TsW8, TsW5, TsW11) and IgGs (TsH12 and TsG10) were added at a concentration of 0.5ug/mL, 100uL/well and incubated at 37°C for 2 hours. After washing, HRP-conjugated goat anti-mouse IgM (Jackson Labs, catalogue number 115-035-075) and goat anti-mouse IgG (Jackson Labs, catalogue number 115-005-003) were added, respectively, at a dilution of 1:20,000, 100uL/well and incubated at 37°C for 45 minutes. Plates were washed, developed with 1-Step Ultra TMB-ELISA substrate solution (Thermo Scientific, cat no. 34029), and reaction stopped (2N H2SO4) at 10 minutes.

### Sample preparation

Patient samples were prepared by adding 150 uL of sample (CSF, plasma, or serum) to an equal volume 5% trichloroacetic acid (TCA) vortexed and incubated at room temperature for 5 minutes. Following vortexing and centrifugation for 5 minutes at 12,000 g, 150 uL were neutralized with an equal volume of Neutralizing Buffer (NB) (1M Tris buffer, pH 8) and vortexed.

### TsG10 (and TsH12)-polyclonal Capture ELISA protocol

TsG10 IgG (and in preliminary experiments TsH12 IgG) in PBS was used to coat 96-well microtiter plates (Immulon 4 HBX, Thermo Scientific) at a concentration of 0.5ug/ml. Coated plates were sealed and stored at 4°C for up to 1 month. Plates were washed 6 times with Wash Buffer (0.025% Tween-20, PBS), blocked for 45 minutes at 37°C with 300 uL/well Blocking Buffer (PBS, 0.05% Tween-20, 5% BSA) and washed again. CSF, plasma, and serum samples were prepared with TCA and NB as above. Standards (Tc Ag serially diluted) or prepared sample were added to wells in duplicate and plates were incubated at 37°C for 45 minutes. Plates were washed and 2.4ug/mL rabbit anti-*Taenia* polyclonal sera was added to each well and plates were incubated at 37°C for 45 minutes. After washing, anti-Rabbit IgG Fc-specific antibodies conjugated to horseradish peroxidase (HRP) (Jackson ImmunoResearch Laboratories, Inc., Code 111-005-046) diluted 1:20,000 was added to each well and incubated at 37°C for 45 minutes. Plates were washed, and 100 uL of 1-Step Ultra TMB-ELISA substrate solution (Thermo Scientific, cat no. 34029) was added to each well, and the plates were incubated for 10 minutes at room temperature in the dark. Following the addition of 2N H2SO4, the optical density (OD) was measured at 450 nm with a reference wavelength of 600–650 nm using a SpectraMax i3 Microplate Reader (Molecular Devices) using the SoftMaxPro 6.5.1 software program.

### TsG10-Biotinylated TsG10 (TsG10-TsG10*bt) Capture ELISA protocol

TsG10 recombinant IgG was biotinylated using the EZ-Link NHS-PEG Solid Phase Biotinylation Kit (Thermo Fisher Scientific cat no. 21450) per manufacturer’s instructions. The product was stored at 4°C in an amber microcentrifuge tube until used. Plates were coated with TsG10, washed, and blocked as above. After incubation with samples or standards, the plates were washed and TsG10-bt* was used at a concentration of 2.4ng/mL. Following a 1-hour incubation at 37°C and washing, 50 uL of HRP-conjugated Streptavidin diluted 1:50,000 was added to each well and incubated at room temperature for 30 minutes. After washing, 100 uL of 1-Step Ultra TMB was added. After 10 minutes, 2N H2SO4 was added to each well. Within 10 minutes of stopping the reaction, the OD of each well was measured at 450 nm with a reference wavelength of 600–650 nm.

### ApDia In vitro diagnostic kit protocol

Steps for the ApDia *In vitro* diagnostic kit for Cysticercosis Ag ELISA followed the manufacturer’s instructions with the following modifications: 1) Kit TCA Solution was replaced with 5% TCA and 2) Kit Neutralization Buffer (NB) was replaced with 1M Tris buffer, pH 8. The data supporting the interchangeability of these reagents is presented in S1 Fig.

### Serum and plasma comparisons

Serum and plasma samples were compared to assess whether these sample types were interchangeable and demonstrated no more variability than well-to-well variation (S1 Table).

### Defining the cutoffs for positivity

The signal-to-noise ratios (SNRs) from 56 samples (21 CSF, 7 plasma, 28 serum) known to be antigen positive by a commercial assay were compared to those of samples from patients/volunteers without NCC (65 healthy control sera, 25 CSF samples from uninfected subjects) TsG10-TsG10*bt and interpreted using ROC curve analysis (GraphPad Prism v9.0). Eight serum samples (ClinicalTrials.gov Identifier NCT00001645) from patients with active (i.e. WHO CE stage 1–3) *E*. *granulosus* disease (6 liver, 2 lung cysts) were processed and run on the TsG10-polyclonal and TsG10-TsG10*bt assays as described above to assess for potential cross reactivity.

### Comparison of TsG10-based assays to commercially available antigen assay

One hundred sixty-nine samples (plasma, serum, CSF) were tested using ApDia, TsG10-TsG10*bt in parallel, each with the same standard curve. TsG10 assay results were determined to be positive or negative based on a ROC-established cutoff (see Results), and concentrations of positive results were extrapolated from the standard curve. Samples run on the ApDia assay were determined to be positive per manufacturer’s instructions, and positive samples were quantified based on extrapolation off the same standard curve.

### Testing of pre- and post- treatment plasma and CSF samples

Well-characterized paired plasma and CSF samples from patients with extra-parenchymal NCC (enrolled on NCT00001205) while disease was active and following cure with active clinical and radiological follow-up of at least 3 years were subjected to testing on each TsG10 platform.

### Plate to plate variability of interpolated antigen concentrations

On each ELISA plate contained high and low positive controls of Tc Ag alongside the same 8 point standard curve described above. Controls were run in parallel on 10 different ELISA plates run on separate days. Control concentration was interpolated off the standard curve and coefficient of variation was calculated by taking the standard deviation across 10 replicates divided by the mean.

### Testing of urine

Thirteen urine samples taken from patients with known current or history of NCC were tested using the TsG10-polyclonal and TsG10-TsG10*bt assays and compared to the ApDia test. Plasma samples obtained from the same patient on the same day were run in parallel. Urine was not processed with TCA or NB but instead applied directly to antibody-coated plates.

### Statistical analysis

Unless other specified, geometric means were used as measures of central tendency. Nonparametric comparisons of group means were made using a Mann-Whitney U test and Wilcoxon Signed rank was used for paired samples. A p-value of less than 0.05 was considered statistically significant for all tests. ROC curve 95% confidence intervals were calculated with the Wilson/Brown method. Correlation analysis was performed using non-parametric Spearman correlation with a two-tailed P value. All statistics were performed using GraphPad Prism Version 8.3.

## Results

### Antibody selection and adaptation

**Fig 1:**
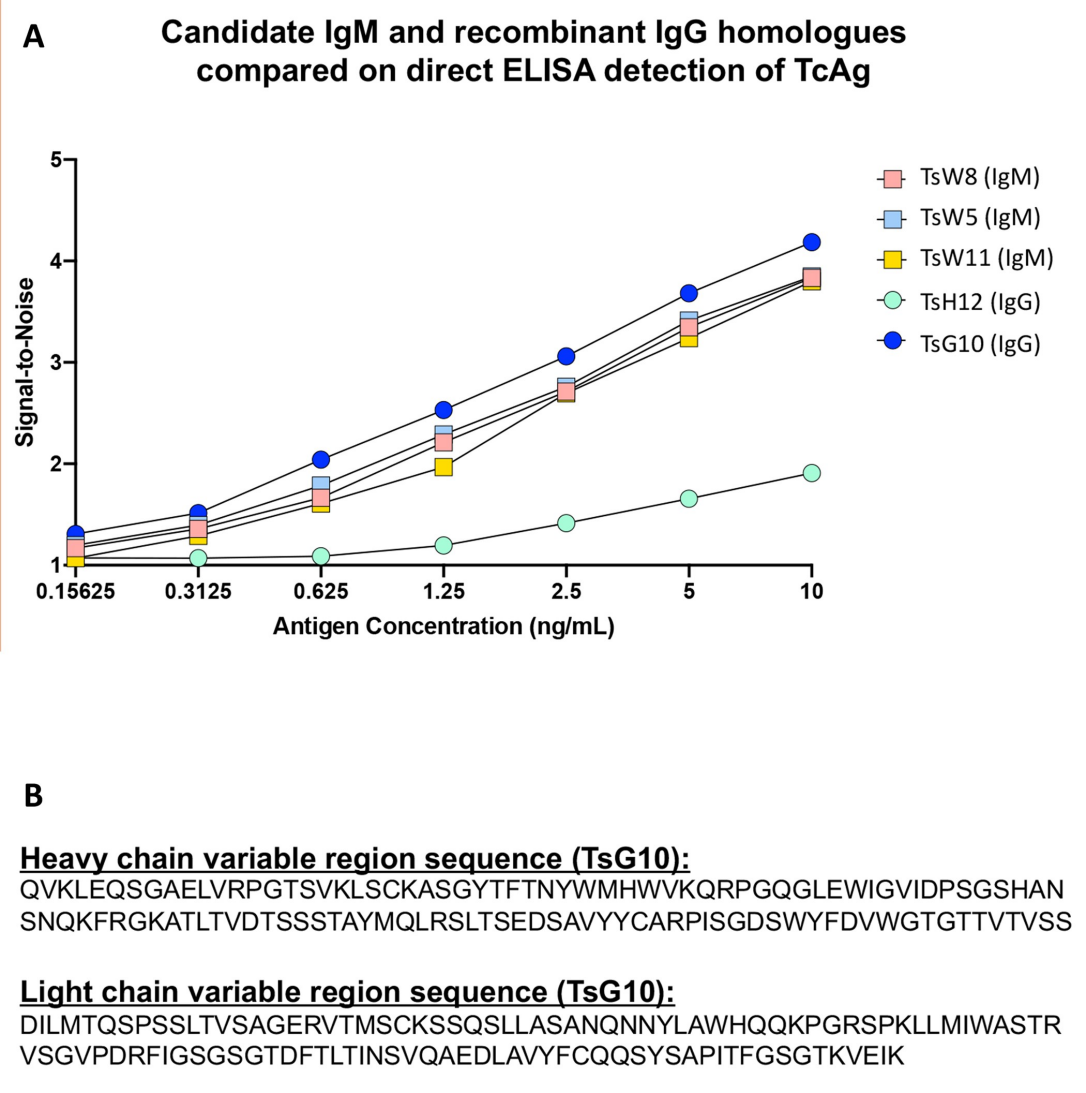
Antibody selection and adaptation. A) Comparison of various potential IgG and IgM antibodies for the detection of *Taenia* antigens. Direct ELISA coated with two-fold serial dilutions of purified *T*. *crassiceps* antigen (Tc Ag). Various IgG and IgM antibodies were used for detection and the S/N ratio is shown. TsG10 is the related IgG of TsW5, while 11H12 is the related IgG of TsW11. B) Complete heavy and light chain variable region amino acid sequences used for construction of the TsG10 IgG antibody.

The three monoclonal IgM antibodies, TsW5, TsW8, TsW11, previously described [7] and the derived recombinant IgGs, TsG10 and TsH12, were used in direct ELISAs to compare the signal-to-noise (S/N) in detecting a range of Tc Ag (Fig 1A). Over the entire dilution series the S/N for the IgM TsW5, and its IgG counterpart, TsG10, were the highest. The second unique IgG, 11H12, had the lowest overall S/N over the dilution curve. Given TsG10 had the higher S/N of the two IgG antibodies, this was carried forward with further experiments. The amino acid sequences of the variable and light chains for TsG10 are in Fig 1B, and for TsH12 are found in S2 Fig.

### Defining limits of the assays

**Fig 2:**
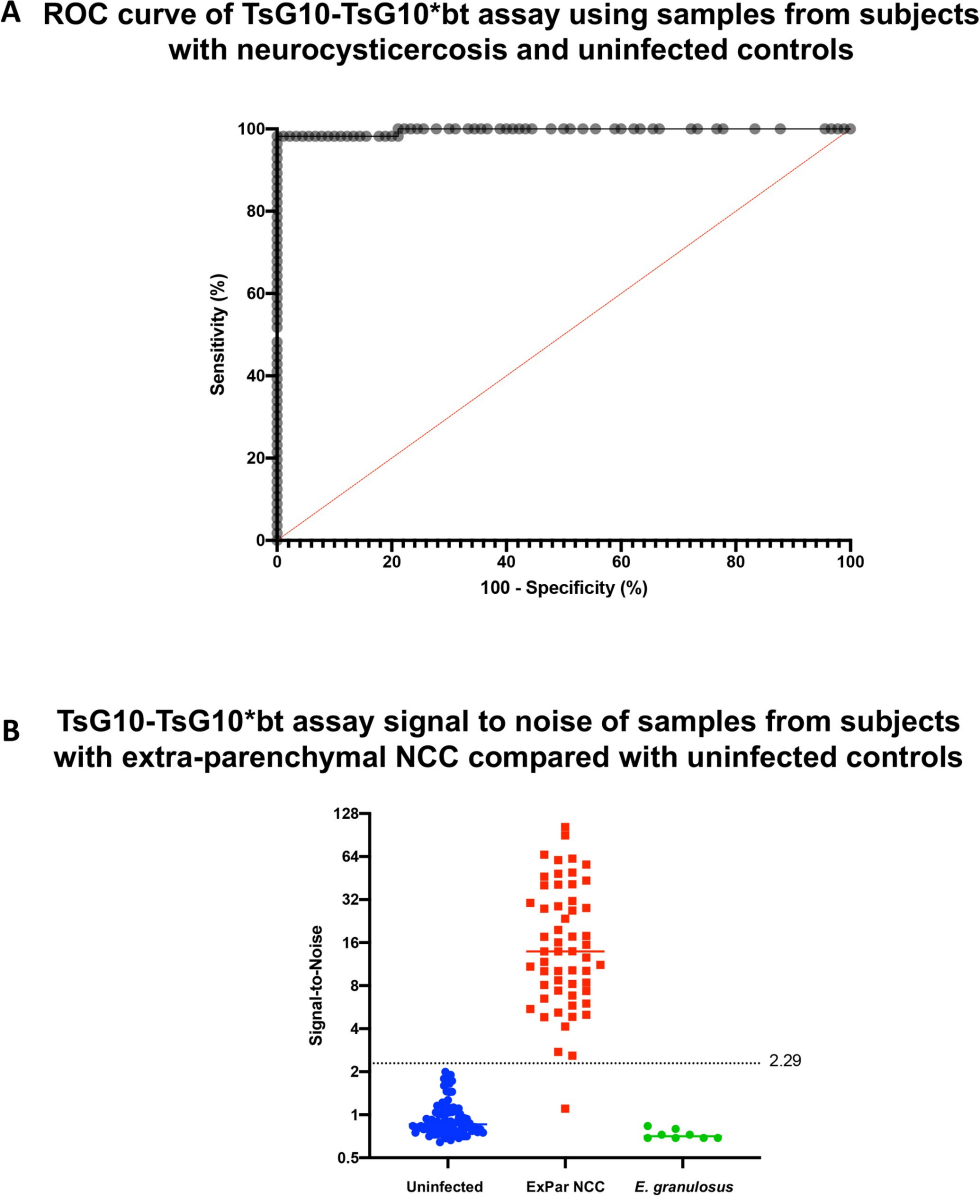
TsG10-TsG10*bt assay determination of the assay cutoff. A) Receiver operating characteristic (ROC) curve of the S/N results testing 90 uninfected control serum/plasma/CSF samples and 56 serum/plasma/CSF samples from patients with known active SANCC and a positive antigen result by a comparator assay (see Materials and Methods). B) Using the Wilson/Brown method, while maximizing sensitivity and specificity, the S/N threshold for positivity was chosen to be >2.29 (line shown) plotted alongside the sample S/N results used for ROC curve construction shown in (A). Also shown are the S/N from serum samples from patients with active *E*. *granulosus* infection.

To determine the limits of detection for each new TsG10 ELISA platform, healthy control blood bank samples and known uninfected CSF samples were compared to plasma, serum, and CSF samples from patients with active extra-parenchymal NCC. Thus, while maintaining a 100% specificity ROC curve demonstrated that at a signal-to-noise cutoff of 2.29, the TsG10-TsG10*bt assay missed only 1of 56 active NCC samples that were positive by a comparator method (Fig 2A and 2B), with a calculated sensitivity of 98.2% (95% CI 90.55%-99.91%). The TsG10-polyclonal assay has a slightly lower sensitivity of 93% at 100% specificity using a S/N cutoff of 2.747, and all further examination of this assay can be found in the Supplemental Data (S2 and S3 Figs, S2 Table). Examination for cross reactivity during *E*. *granulosus* infection was sought on both assays. Serum from 8 patients with active (CE1-3) *E*. *granulosus* disease was tested and none were positive by either TsG10 assay.

### Comparison of TsG10-based assays to commercially available antigen assay

**Fig 3:**
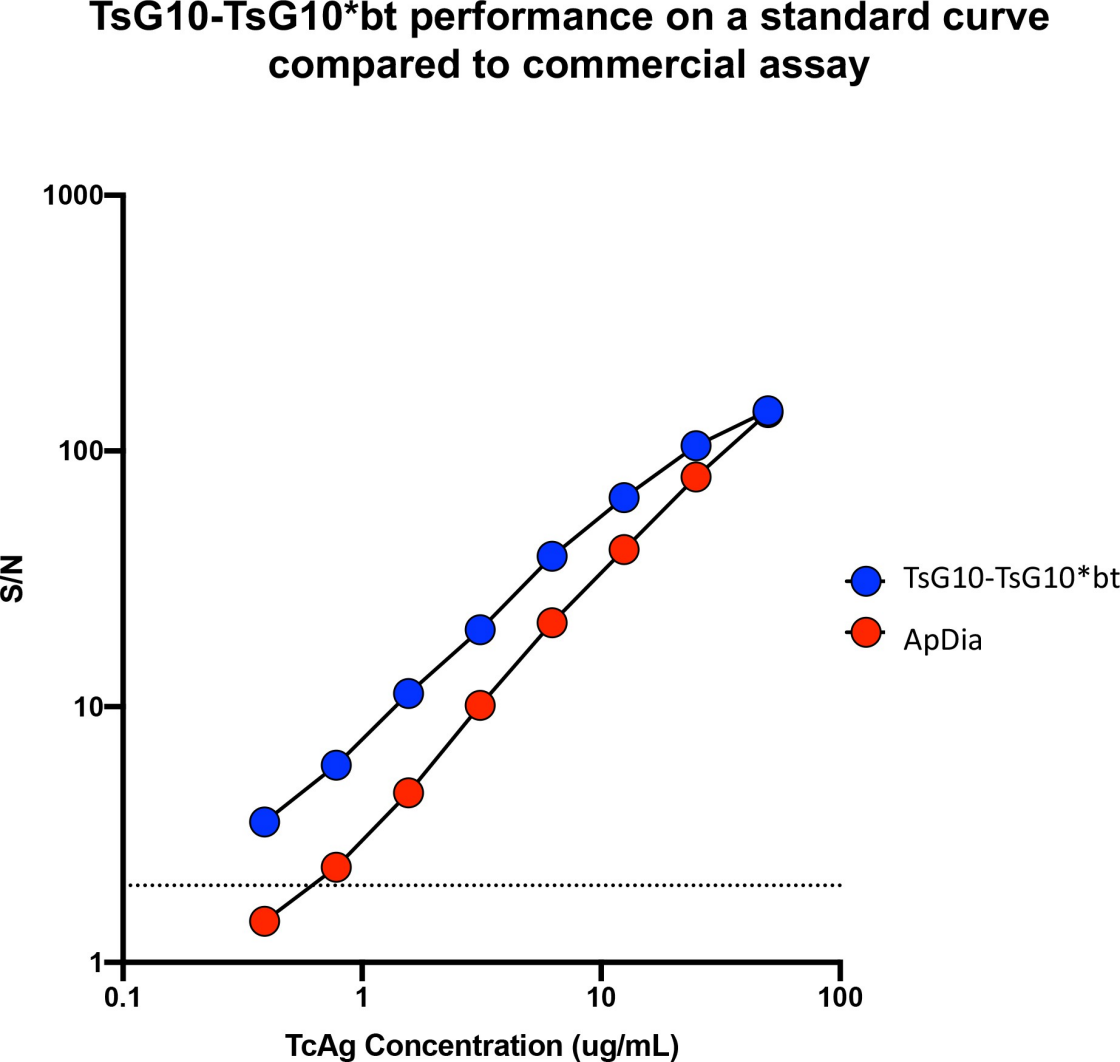
Tc Ag detection of standards using the TsG10 and comparator assays over a dilution series, shown as S/N ratios. This standard curve was used so that Ts Ag concentrations in patient samples could be interpolated.

**Fig 4:**
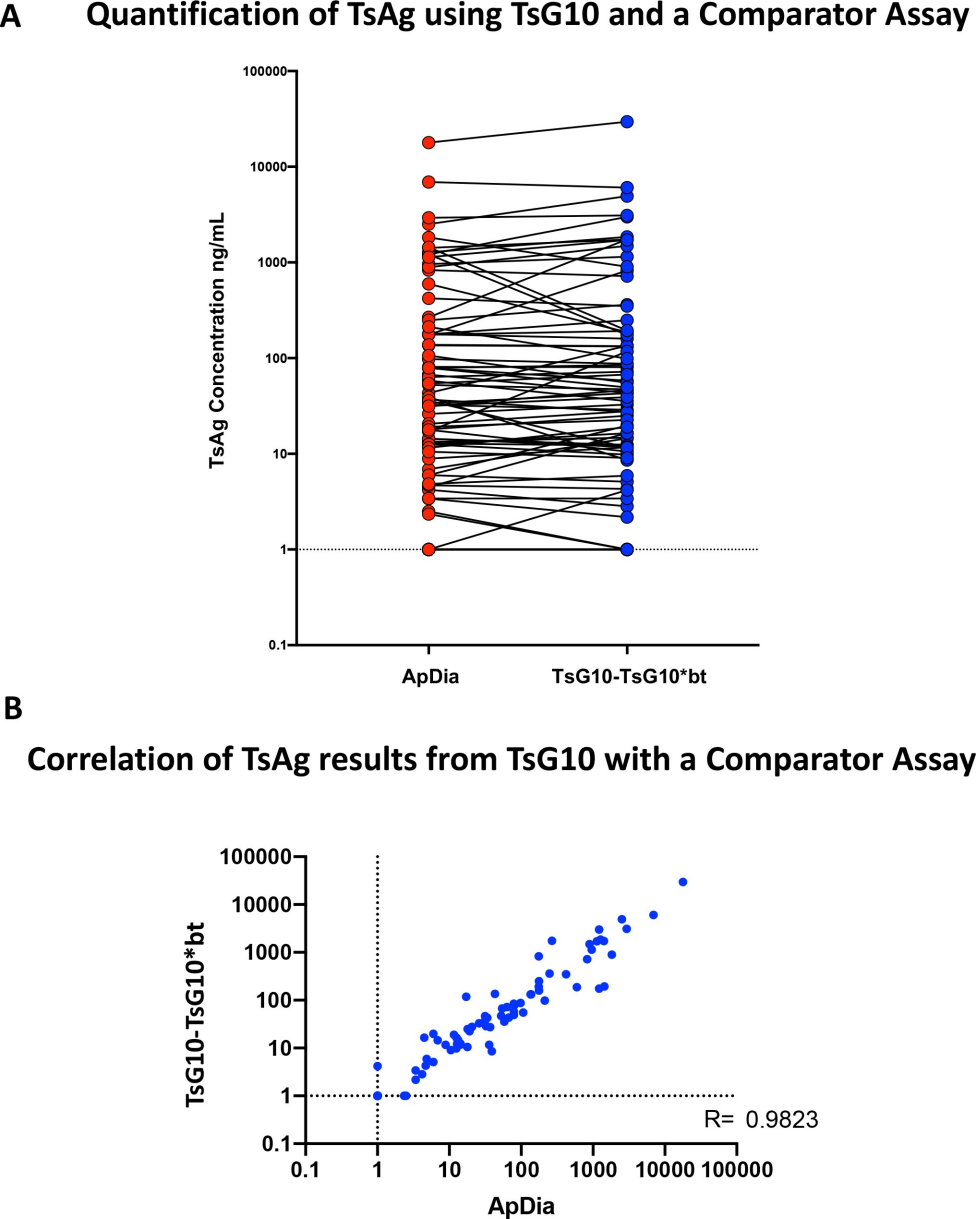
A) Serum, plasma, and CSF samples from patients with known SANCC, but at various stages of treatment (including cured) were tested by the TsG10-TsG10*bt and the comparator assay, ApDia. Both assays were run with the same standard curve and quantitative results interpolated from the standard curve. Data are antigen concentrations +1 (ng/ml). B) Spearman Correlation analysis using the sample results from (A).

Using the identical standard Tc Ag curve (Fig 3), values for 169 samples of plasma, serum, and CSF from patients with proven NCC were assessed both using the TsG10-TsG10*bt assay and the commercially available Apdia assay (Fig 4A). As can be seen in Fig 4B there was an extremely strong correlation between the values obtained in the ApDia and those obtained in TsG10-TsG10*bt assay (r = 0.9823, p < 0.0001).

### Changes pre- and post- treatment

**Fig 5:**
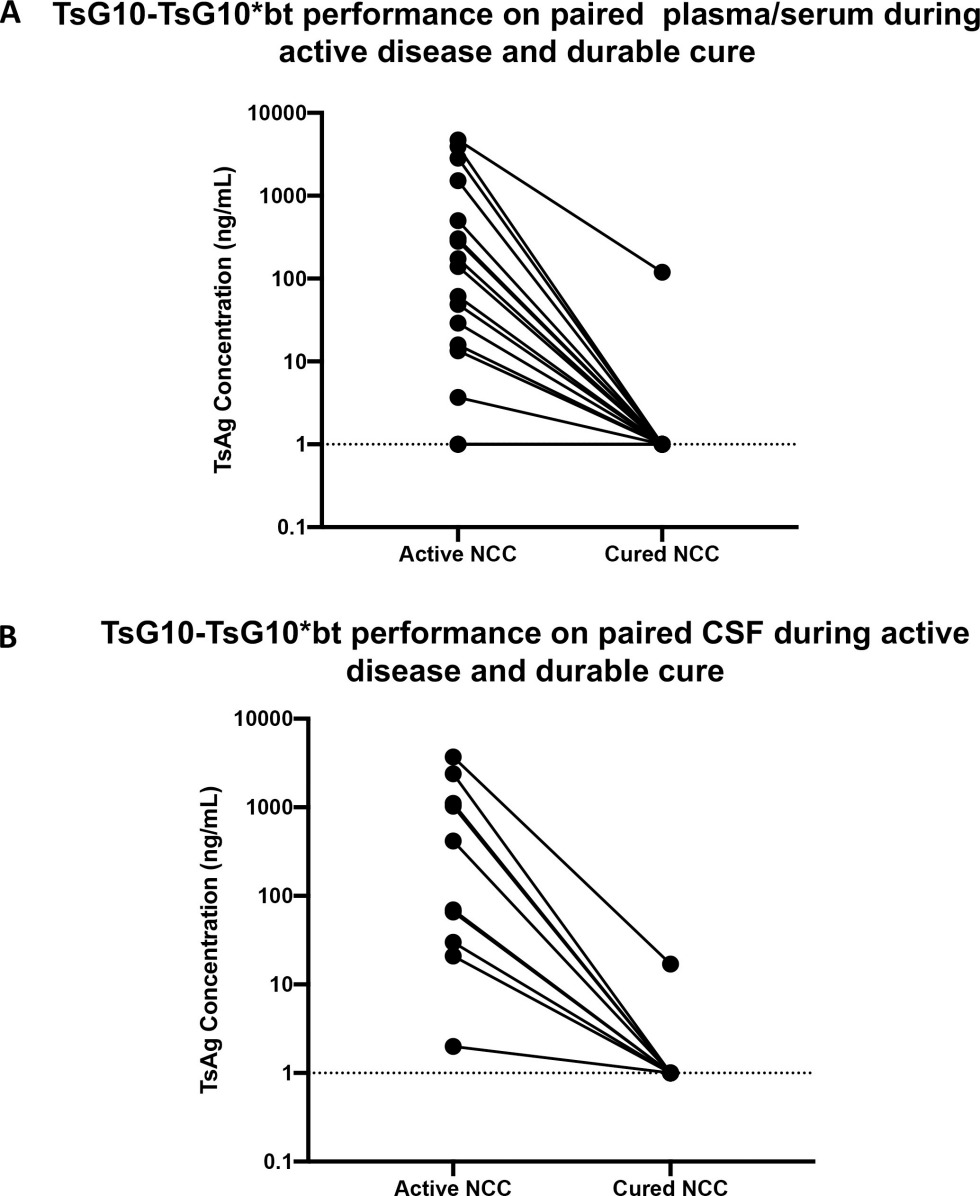
Paired plasma/serum and CSF sample Ts Ag results in active and cured extra-parenchymal NCC (A) Twenty paired plasma/serum and (B) 10 paired CSF samples from patients with active extra-parenchymal NCC during active disease (early in treatment) and at the time of cure. Data are antigen concentrations +1 (ng/ml).

Paired samples (20 plasma/10 CSF) taken at time of diagnosis with active subarachnoid racemose disease and at least 3 years following cure (based on clinical and radiologic follow-up) were tested in the Mab-based antigen detection immunoassay (Fig 5). As shown, 19/20 (95%) samples from patients with active disease were Ag positive in the plasma at baseline; all but 1 became Ag negative (P<0.0001). All 10 (100%) of CSF samples from patients with active disease were positive for Ts Ag at baseline, with 9/10 becoming negative following clinically defined cure (P = 0.002).

### Plate to plate variability of extrapolated antigen concentrations

High and low controls were run on each of 10 sequential TsG10-TsG10*bt assays. The high control averaged 36.9 ng/mL with a coefficient of variation of 14.6%, and the low control averaged 1.6 ng/mL with a coefficient of variation of 9%.

### Ts Ag detection in urine samples

Only 15 urine samples were available for testing in patients with extra-parenchymal NCC, and they came from all stages of infection, and many were cured. Of the 7 that had positive TsG10-TsG10*bt antigen results from the serum, 4 were also detected in urine (Table 1).

**Table 1:** Detection of Ts Ag in the urine by TsG10 assay compared to concurrent TsG10 serum/plasma antigen detection results in patients with extra-parenchymal NCC in various stages of treatment.

Serum/Plasma	Urine
23	15.158
8.3	0
0	0
0	0
0	0
0	0
38	4.201
0	0
14	28.992
2.8	0
0	0
0	0
146	25
5.5	0
0	0
Correlation Coefficient	0.81, p = 0.0007

## Discussion

While two clinical *T*. *solium* antigen detection assays have already been well described in the literature [4, 5] the worldwide access to these tests is limited by possessing the hybridoma or having access to the hybridoma product. Additionally, over time hybridomas can experience genetic drift, resulting in antibody variation. The B158/B60 ELISA, the comparator assay in this study, has been applied to testing in human and porcine samples. In patients with parenchymal NCC, positivity has been shown to correlate to burden of disease, and the S/N threshold for positivity can be set adjusted to have a high sensitivity for active parenchymal disease in the setting of epilepsy [8]. While other antigen assays are known to cross react with the other porcine pathogen *Taenia hydatigena*, the negative predictive value of this test in pigs has been shown to be 98% [9]. Cross reactivity to *T*. *hydatigena* porcine infection was not assessed in the current study but would be valuable to determine in future studies.

Here, we describe a recombinant monoclonal-based assay with sensitivity and specificity for detecting Taenia antigen in the CSF and peripheral blood essentially equivalent to this previously described assay [5]. Moreover, the relative antigen quantification between the two assays is highly correlated (r = 0.98). The assay is robust and highly reproducible, with minimal plate-to-plate variation and no demonstrable difference between using serum vs plasma. Cross reactivity was assessed since occasionally the cysts of *E*. *granulosus* can present similarly in appearance to NCC [10]. While a small sample size was used, all serum/plasma samples from patients with active cystic echinococcosis tested negative by this assay.

There are significant challenges in diagnosing and treating patients with extra-parenchymal NCC. Recurrent and protracted disease in the racemose/subarachnoid phenotype and the unreliability in following clinical and radiologic changes [2]. Despite the high morbidity [11] and mortality [12] reported in this patient population, our group has previously demonstrated the utility of using a comparator assay to determine durable cure, which enables the safe discontinuation of treatment [13]. Here we demonstrate that TsG10-TsG10*bt was 95% sensitive in the serum and 100% sensitive in the CSF in detecting active extra-parenchymal disease in our patient population. Moreover, in those with durable cure the serum antigen converted from positive to negative in 95% of patients and in the CSF of 9 of 10 patients. This assay adds to the armamentarium of available tests that can be done to follow response to treatment in extra-parenchymal NCC [3, 14].

Others have noted the ability to detect Taenia antigens in the urine of patients with NCC [15]. Urine antigen levels have been observed to correlate with viable cyst burden [16], and detection has been used as a population screening tool for subarachnoid disease in Peru [17]. Here we also found a good correlation between serum/plasma and urine antigen levels in the small sample size we examined (r = 0.81). Sensitivity was diminished when the serum/plasma antigen level was low (less than 10ng/mL). This assay could be similarly utilized to screen for heavy infections in large populations. Urine requires no processing to precipitate out serum proteins, so mass screening utilizing this method has the advantage of faster times to assay completion, and of course does not require needle sticks or specialized equipment as is the case with blood draws.

Access to recombinant antibody technology and the instrumentation needed for ELISAs are limitations to the use of this assay. Our hope is that the increased accessibility to the required reagents involved in this assay compared to previous *T*. *solium* antigen assays will broaden clinician and researcher access to this extremely useful tool. Moreover, given the highly scalable and consistent nature of monoclonal antibodies produced through mammalian cell line expression, optimally it could be converted into a lateral flow assay that could be mass produced for point-of-care testing. Whether used for population screening as a urine test in the field, a tool for healthcare workers in under-resourced areas, or even healthcare workers in high volume high-resourced settings, a widely available tool for diagnosis and disease monitoring in NCC would be valuable for clinicians and researchers.

## Supporting information

S1 Fig Comparison of optical densities obtained processing serum samples with the ApDia kit (A) neutralization buffer (NB) versus in-house neutralization buffer, and (B) Trichloroacetic acid (TCA) versus in-house TCA. Serum samples from known antigen positive and negative samples were processed in parallel by ApDia kit and in-house reagents with comparable results.
(TIF)

S2 Fig Amino acid sequence of TsH12 heavy and light chain variable regions. (TIF)

S3 Fig Comparison of the signal-to-noise results across a 8 point standard curve using *Taenia crassiceps* antigen with 2-fold dilutions between the TsG10-Polyclonal, TsG10-TsG10*bt, and ApDia assays. (TIF)

S1 Table Comparison of plasma versus serum optical density results (A) Individual replicate results and calculations for plasma versus serum coefficient of variation and well-to-well coefficient variation for both polyclonal and monoclonal (TsG10-TsG10*bt) assays. Serum and plasma samples drawn at the same time in patients with a variety of different levels of antigen positivity. Serum and plasma samples were processed in duplicates and in parallel and run on the same date and results between sample type compared to variability between duplicates. (B) Averages of individual replicates demonstrates the variability between duplicates (well-to-well) is comparable (5% and 7% polyclonal and monoclonal, respectively) to the variability between the use of serum versus plasma (5.2% and 5.7% polyclonal and monoclonal, respectively).
(TIF)

S2 Table Detection of TsAg in the urine by TsG10-Polyclonal assay compared to concurrent ApDia serum/plasma antigen detection results in patients with extra-parenchymal NCC in various stages of treatment, including the correlation coefficient. (TIF)
