# Supplementary figures and images for: A recombinant monoclonal-based Taenia antigen assay that reflects disease activity in extra-parenchymal neurocysticercosis
Source: PLoS Negl Trop Dis. 2022 May 26;16(5):e0010442. doi: 10.1371/journal.pntd.0010442 (PMC9176767; doi:10.1371/journal.pntd.0010442)

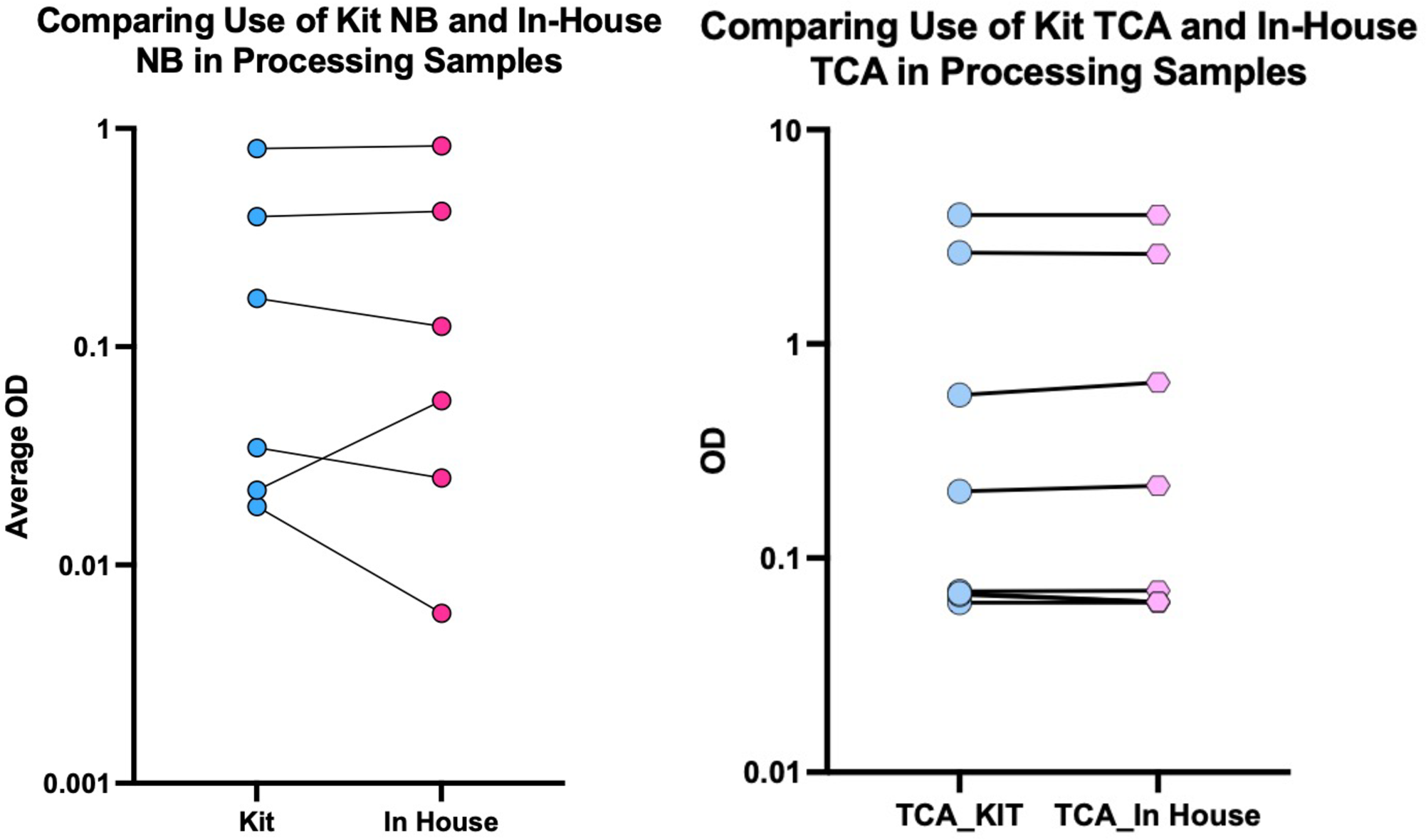

Supplement: S1 Fig — Comparison of optical densities obtained processing serum samples with the ApDia kit (A) neutralization buffer (NB) versus in-house neutralization buffer, and (B) Trichloroacetic acid (TCA) versus in-house TCA. Serum samples from known antigen positive and negative samples were processed in parallel by ApDia kit and in-house reagents with comparable results. (TIF) [file pntd.0010442.s001.tif]

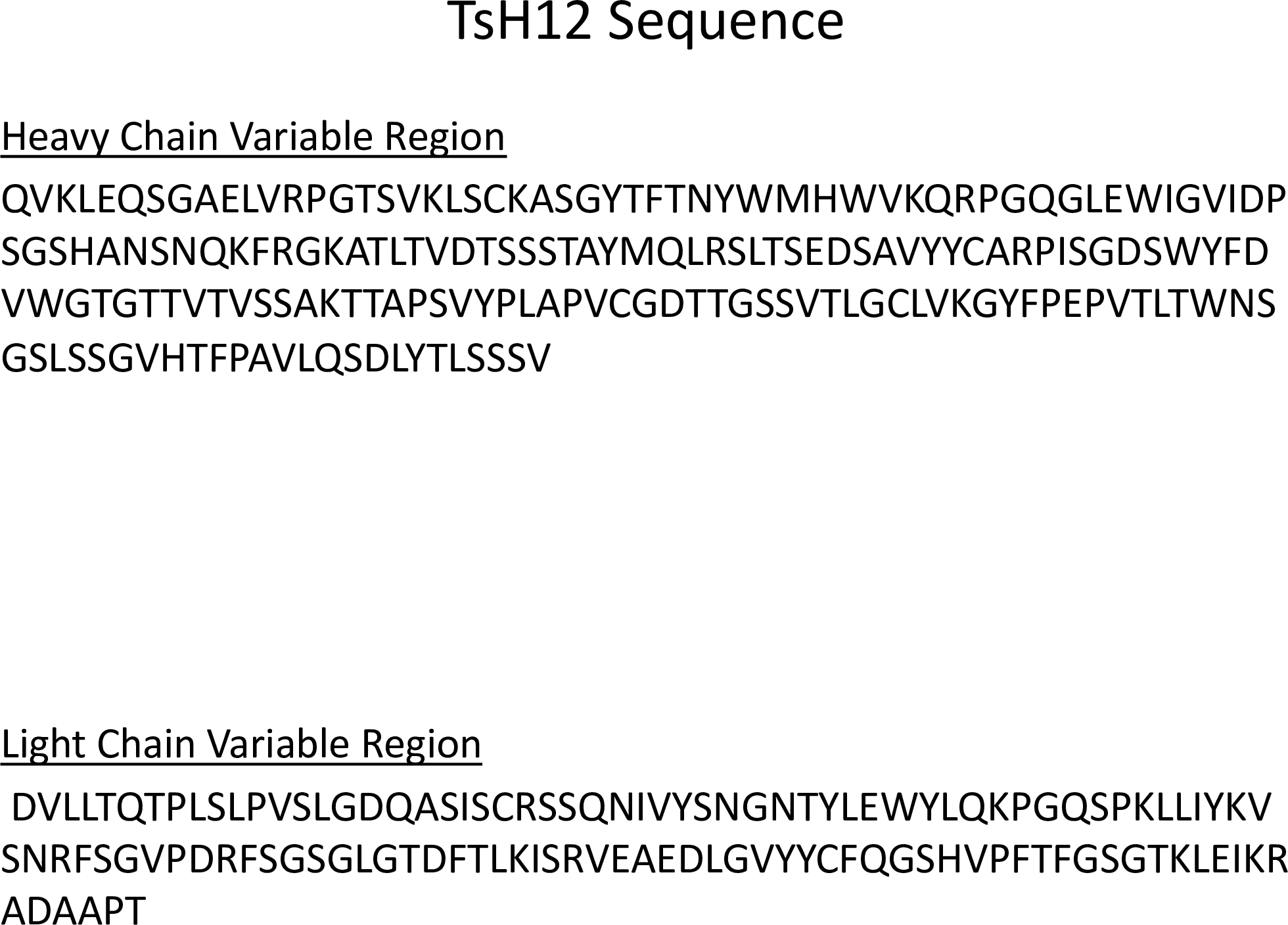

Supplement: S2 Fig — (TIF) [file pntd.0010442.s002.tif]

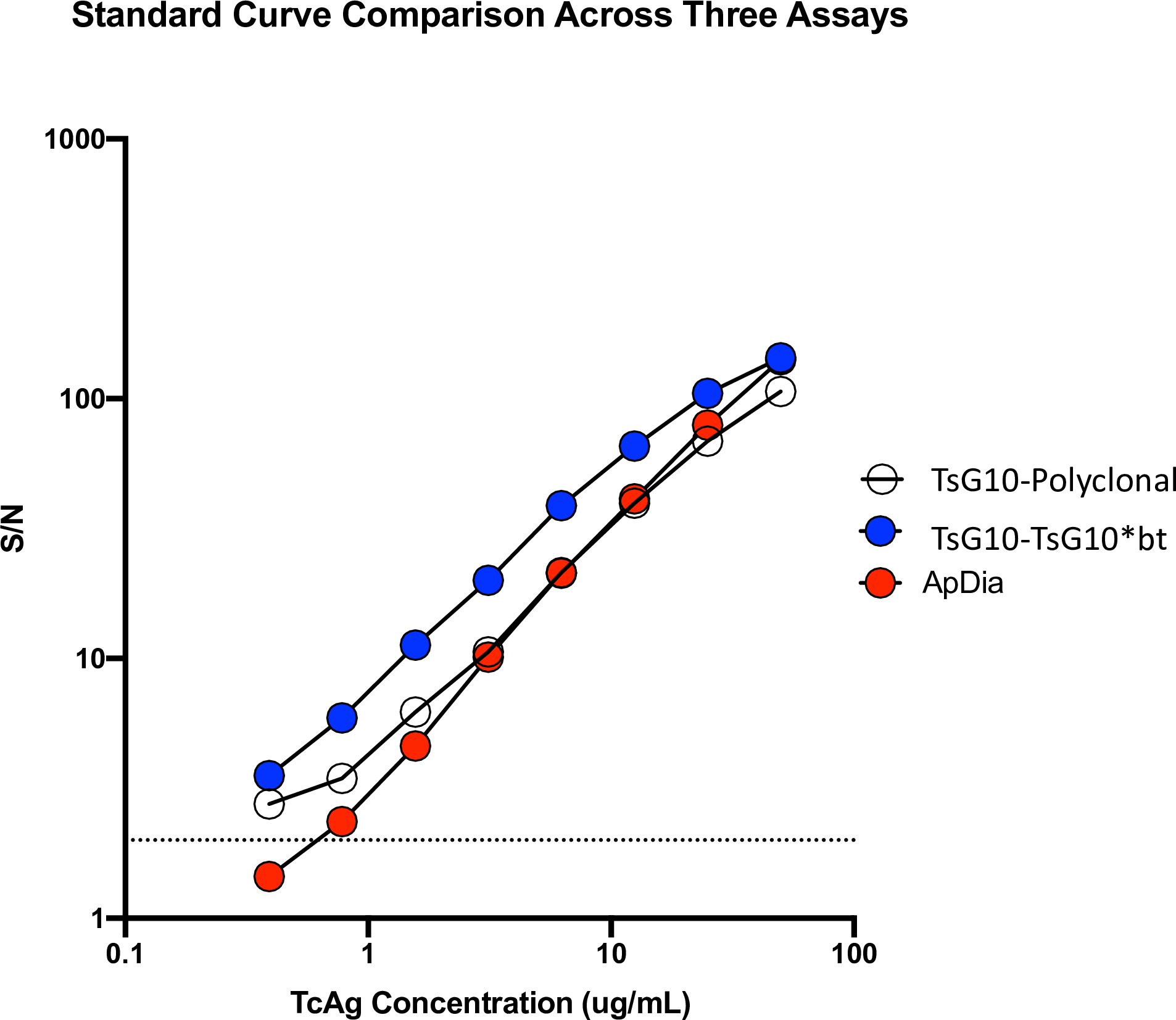

Supplement: S3 Fig — (TIF) [file pntd.0010442.s003.tif]

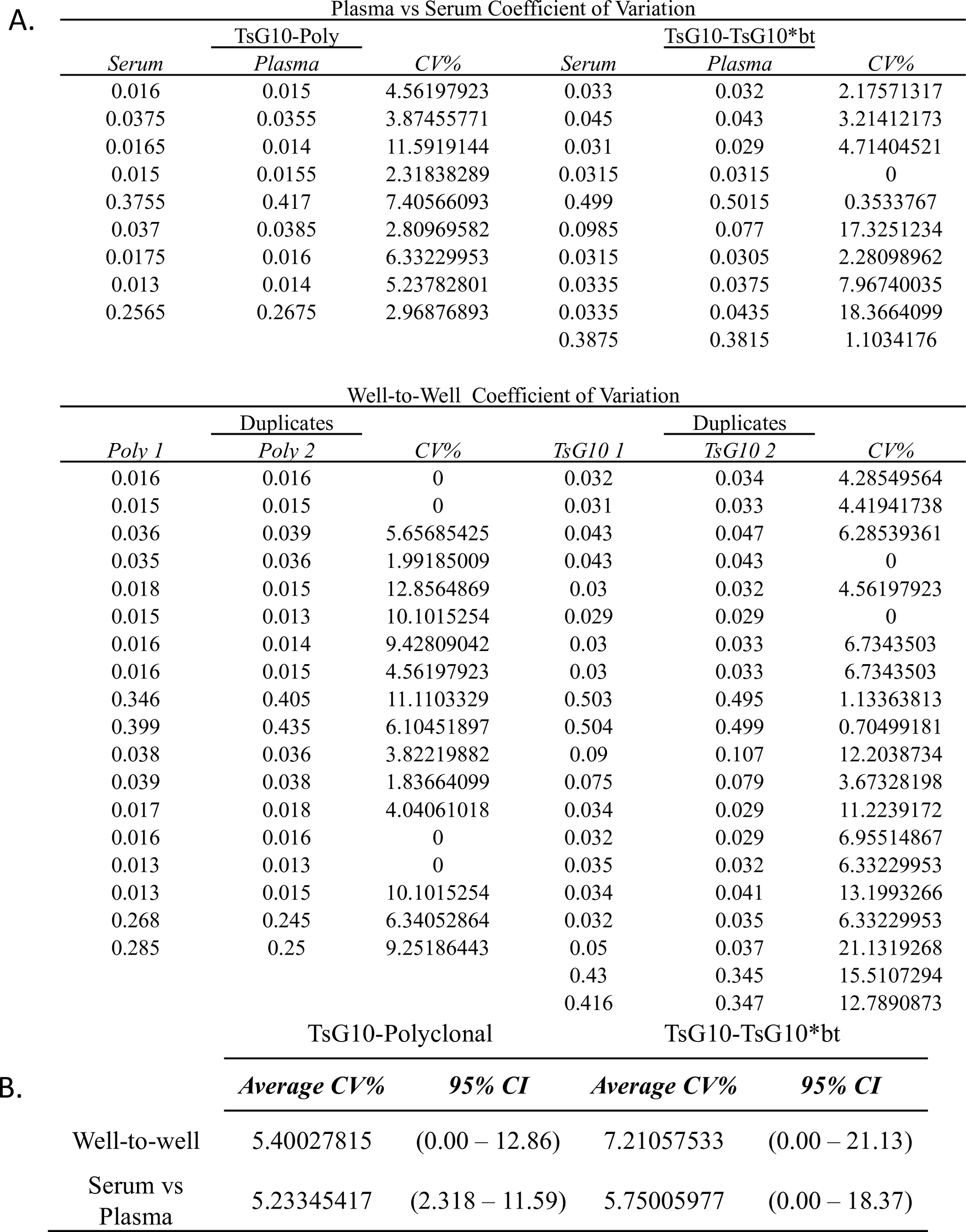

Supplement: S1 Table — Comparison of plasma versus serum optical density results (A) Individual replicate results and calculations for plasma versus serum coefficient of variation and well-to-well coefficient variation for both polyclonal and monoclonal (TsG10-TsG10*bt) assays. Serum and plasma samples drawn at the same time in patients with a variety of different levels of antigen positivity. Serum and plasma samples were processed in duplicates and in parallel and run on the same date and results between sample type compared to variability between duplicates. (B) Averages of individual replicates demonstrates the variability between duplicates (well-to-well) is comparable (5% and 7% polyclonal and monoclonal, respectively) to the variability between the use of serum versus plasma (5.2% and 5.7% polyclonal and monoclonal, respectively). (TIF) [file pntd.0010442.s004.tif]

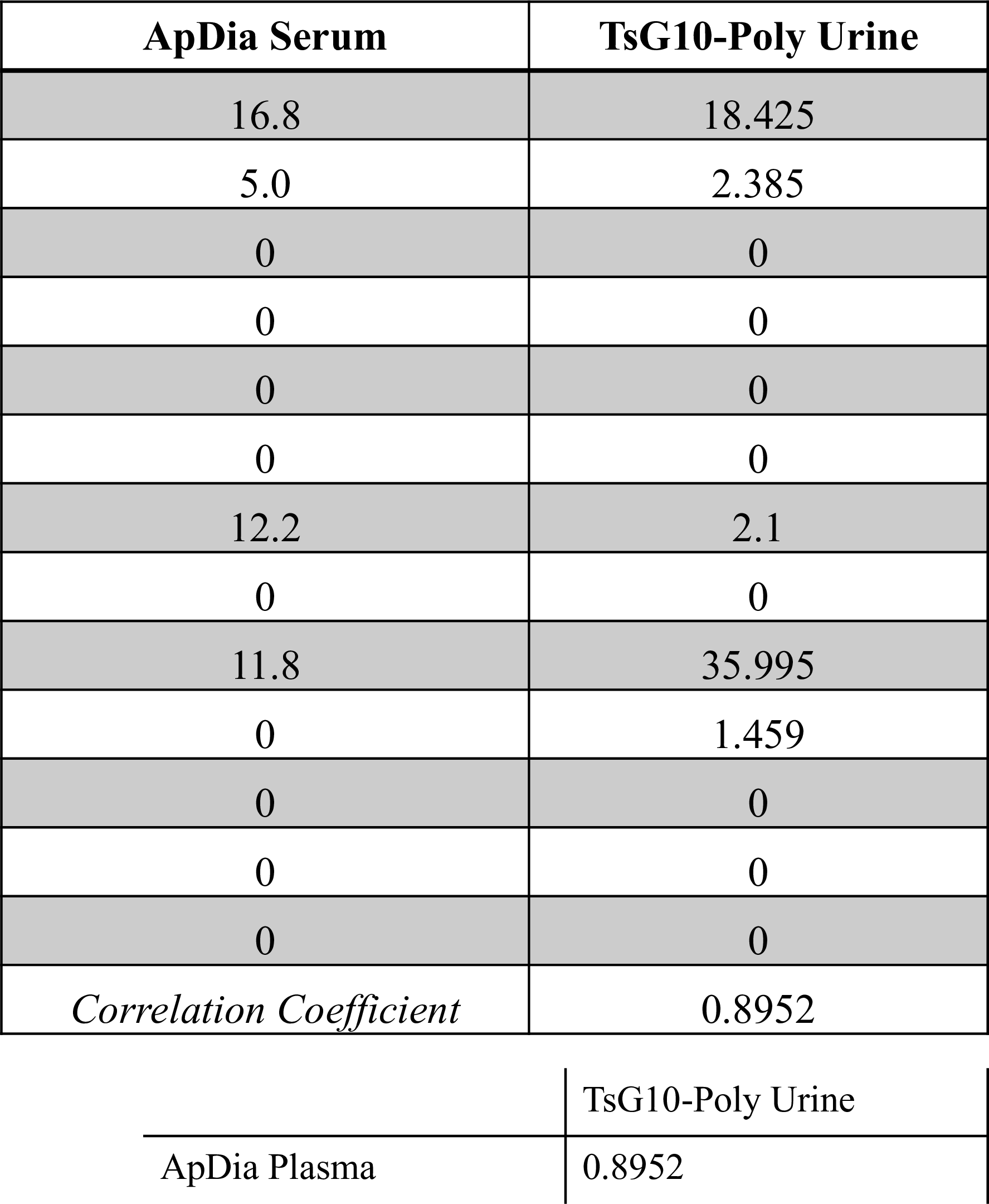

Supplement: S2 Table — (TIF) [file pntd.0010442.s005.tif]
